# Association of Sporadic and Genetic Parkinson’s Disease with Cancer: Insights from the PPMI Cohort

**DOI:** 10.3390/genes16111302

**Published:** 2025-11-01

**Authors:** Evangelos Sfikas, Christos Koros

**Affiliations:** 1st Department of Neurology, Eginition Hospital, National and Kapodistrian University of Athens, 15772 Athens, Greece; vasfikas@gmail.com

**Keywords:** Parkinson’s disease, cancer, neoplasm, genetic, alpha-synuclein, glucocerebrosidase

## Abstract

Background/Objectives: Prior studies have reported a complex interplay between Parkinson’s disease (PD) and malignancy. Although patients with PD often present a lower general risk for several types of cancer, some forms—including melanoma—show elevated frequency. The present work aimed to evaluate the occurrence of cancers other than melanoma among individuals with sporadic and genetic PD. Methods: We examined medical histories from 1888 participants with PD and 438 healthy controls (HCs) using the Parkinson’s Progression Markers Initiative (PPMI) dataset, with a focus on neoplastic disease. In cases with positive cancer history, genetic information was additionally assessed [carriers of mutations in the most prevalent PD-related genes were evaluated]. Results: Our results demonstrate that cancer incidence was antecedent to PD diagnosis for the majority of PD patients, while the most common cancer types apart from malignant melanoma were non-melanoma skin cancer and prostate cancer. Conclusions: Regarding genetic PD patients, the most common cancer types in the *LRRK2* and *GBA1* groups were skin cancer and lymphoma, while *PRKN/PARK2* carriers appeared with an overall increased incidence of cancer. No statistically significant results were observed comparing cancer incidence in PD patients to that in healthy control individuals. Interesting results were obtained by dividing the patients by gender, showing increased cancer risk in female PD patients and female *LRRK2* carriers, along with increased breast cancer risk in female PD patients compared to healthy controls.

## 1. Introduction

Parkinson’s disease (PD) is a chronic progressive neurodegenerative disorder characterized by both motor and non-motor system manifestations. It occurs mostly in older individuals, but it can also manifest in younger patients [1]. PD represents the second most common neurodegenerative disease [2]. Other neurodegenerative disorders may mimic PD features. These include Dementia with Lewy Bodies (DLB), Corticobasal Degeneration (CBD), Multiple System Atrophy (MSA), and Progressive Supranuclear Palsy (PSP) [3].

Pathologically, PD is defined by the selective loss of dopaminergic neurons in the substantia nigra pars compacta along with the accumulation of Lewy bodies, which are protein aggregates composed primarily of abnormal alpha-synuclein along with other proteins [4]. These pathological changes may precede clinical signs by decades. Disruptions in protein degradation systems such as autophagy and the ubiquitin–proteasome pathway, together with mitochondrial dysfunction and oxidative stress, contribute to neuronal death [5,6,7,8].

Specifically, alpha-synuclein becomes abnormal and self-aggregates. This aggregated, insoluble form of alpha-synuclein is the major constituent of Lewy bodies. In addition, systems designed for quality control of abnormal proteins, like the ubiquitin–proteasome system and autophagy, become impaired. Other abnormal processes that may play a role in PD are mitochondrial dysfunction or oxidative stress through reactive oxygen species causing neuronal degeneration and cell death [9,10].

Clinically, PD is recognized through tremor, bradykinesia, rigidity, and postural instability as the core motor features. A wide range of non-motor manifestations—including sleep disturbance, autonomic dysfunction, psychiatric changes, sensory symptoms, and cognitive decline—often precede motor onset by years. It is estimated that nearly 90% of patients experience non-motor symptoms throughout the disease course [10,11]. Age is the strongest risk factor, with typical onset between 50 and 60 years. Family history and environmental exposures such as pesticides also play a role. Despite sporadic PD accounting for the majority of cases, familial PD accounts for 5–15% of them. The most common genetic cause of PD consists of *LRRK2* and *GBA1* gene mutations, while other related genes are *SNCA*, *PRKN/PARK2*, and *PINK1* [10,12]. Additional risk factors have been identified, though how they may differentially affect men vs. women is still unclear.

Additional environmental and lifestyle risk factors have been reported, including rural living, agricultural work, exposure to metals, consumption of well water, and higher educational level, although the findings remain inconsistent [11].

Interestingly, a number of studies have suggested that patients with PD may show lower risks for certain cancers, though the mechanisms behind this apparent inverse relationship remain unclear [13,14,15,16,17,18,19,20].

PD is the clinical expression of gradual nigral neuronal cell death with a chronic and generally slow progression. On the contrary, cancer is usually the result of rapid cell proliferation, infiltrating neighboring tissues and distant organs with an aggressive profile. Despite apparently not sharing many similarities, overlapping biological pathways underlie both PD and cancer pathologies, such as activation of immune response mechanisms, chronic inflammation, DNA damage, mitochondrial dysfunction, metabolic shifts, transcriptional dysregulation, autophagy impairment, and alternative splicing [15]. Similarities can be noticed as well in their additional clinical features; both disorders could be triggered by events or exposures much earlier in life, and both cancer and PD clearly develop due to the interaction of genes and environmental factors [18].

One of the most significant factors related to both PD and cancer is age, while smoking is associated with many types of cancer, although it has an inverse association with PD. As far as sex is concerned, female PD patients have been linked to a reduced risk of cancer. Accumulative evidence has shown that PD patients generally have relatively reduced risks of colon, rectal, colorectal, and lung cancer and increased risks of brain cancer and melanoma, while genetic *LRRK2*-G2019S PD carriers have increased cancer risk, particularly regarding brain, breast, colon, and blood cancers. Moreover, *PRKN/PARK2* loci have been related to an overall increased risk of cancer. Previous studies have shown a positive association between genetically undetermined PD (GU-PD) and *GBA1*-PD with malignant melanoma. Last, but not least, many studies emphasize the role of the synuclein protein family in cancer, with α-synuclein being expressed in many types of cancer and participating in tumor-promoting mechanisms [15,17,18,19,20].

The Parkinson’s Progression Markers Initiative (PPMI) is an international observational program designed to follow the natural course of Parkinson’s disease. Backed by the Michael J. Fox Foundation and multiple partners, the study involves more than 50 research centers worldwide. Its goal is to track both motor and non-motor symptoms over time, with particular attention to cognitive decline. The PPMI also compiles diverse biomarkers, such as brain imaging, biological samples, genetic information, and digital health data. The primary objective is to discover reliable markers of disease progression that can accelerate the testing of new therapeutic strategies. Participants include prodromal cases, newly diagnosed untreated patients, carriers of genetic variants linked to PD, and healthy controls.

We previously assessed the prevalence of malignant melanoma in idiopathic and genetic PD patients participating in the PPMI cohort [17]. In the present study, we aimed to evaluate the prevalence of any type of cancer other than melanoma in patients with idiopathic and genetic types of PD and provide insights regarding cancer risk in PD patients. Moreover, another goal was to investigate whether certain types of cancer appear in PD patients (either idiopathic or genetic) in higher frequencies than others. The impact of gender in the association between PD and cancer was also assessed. This was accomplished by deploying the large dataset of the PPMI study.

## 2. Materials and Methods

The PPMI is a prospective, multi-center longitudinal study designed to evaluate clinical characteristics, dopamine transporter (DAT) imaging, and biological markers in individuals with Parkinson’s disease. The eligibility criteria are outlined at www.ppmi-info.org. For PD enrollment, participants were required to have typical motor features, a diagnosis within two years at screening, Hoehn and Yahr stage I–II at baseline, evidence of abnormal DAT imaging, and no history of PD-specific treatment for at least six months before baseline. Both men and women aged 30 years or older at diagnosis were included. Control participants, also ≥30 years of age, were required to have no neurological illness and no exposure to medications affecting dopamine transporter SPECT imaging. Importantly, carriers of rare genetic variants such as *SNCA*, *PRKN*, or *PINK1* were allowed to participate regardless of disease duration.

Participants are assessed over time, with comprehensive visits each year and shorter interim visits every six months. Initial visits collect epidemiological data, while subsequent follow-ups include detailed physical and neurological examinations. Information on past medical history, comorbidities, and current medications is systematically recorded. A broad battery of evaluations—including motor, cognitive, laboratory, and neuroimaging assessments—generates a rich dataset for future analyses.

The study is conducted under strict ethical standards. All participants provided written informed consent. Institutional review boards at 51 clinical centers (listed at ppmi-info.org) approved the protocol. The study adheres to the Declaration of Helsinki and subsequent amendments, with oversight also granted by the Scientific Boards of each site, including Eginition Hospital.

Data for participants with Parkinson’s disease (PD) and healthy controls (HCs) were obtained from the PPMI database (www.ppmi-info.org) on 1 February 2025. In total, records from 1888 PD patients and 438 HCs were reviewed. The analysis focused on past medical history, comorbid conditions, and genetic background, with particular attention to reported cases of cancer. Malignant melanoma was excluded from the present work, as it was analyzed separately in a previous study [17]. Additional demographic variables—including race, sex, and age at the time of PD or cancer diagnosis—were also considered. Genetic data were retrieved for those PPMI participants with a positive history of cancer, specifically carriers of PD-related mutations (carriers of *LRRK2* gene mutations, *GBA1* gene mutations, *SNCA* gene mutations, *PRKN/PARK2* gene mutations, and *PINK1* gene mutations).

Statistical analysis regarding the cancer risks across groups was performed using the chi-square test in MedCalc Statistical Software version 23.0.8 (MedCalc Software Ltd., Ostend, Belgium; https://www.medcalc.org; 2024). Statistically significant results required *p*-value < 0.05. Results were accordingly converted into forest plots.

## 3. Results

In total, 1888 participants with PD were screened, and 171 (9.06%) were found with a positive medical history of cancer of any type, except for melanoma (Table 1, Table 2 and Table 3). The vast majority of them were of white race (97.68%). Most of those patients were of male sex (64.91%) with a mean age at cancer diagnosis of 63.20 ± 11.34 years, while the mean age at PD diagnosis was 66.25 ± 8.52. As a result, cancer incidence was antecedent to PD diagnosis in 70.76% of those patients. The types of cancer appearing with higher prevalence in PD patients were skin cancer (4.40%), prostate (2.38%) and breast cancer (0.85%), lymphoma (0.58%), and urinary tract cancer (0.53%), with other types occurring in lower frequencies of ≤0.5%.

Concerning the healthy control participants (Table 2 and Table 3), we observed that of 438 total individuals, 36 (8.22%) had a positive history of cancer of any type, except for melanoma. As with the PD patients, the vast majority of them were also of white race (97.22%). Most of those patients were of male sex (61.11%) as opposed to female (38.89%). The mean age at cancer diagnosis did not differ much from that observed in PD patients (62.61 ± 11 years). Like for the PD patients, the cancer types with higher prevalence were skin cancer (5.71%), prostate cancer (2.28%), breast cancer (0.68%), and other rare cancers (0.91%), while the remaining neoplasia types occurred in frequencies of 0.23% each.

Additionally, we focused on PD subgroups as defined by the genetic status of these patients. Out of 1523 patients in the PPMI study with idiopathic PD, 143 (9.39%) were found with a recorded history of cancer of any type, other than melanoma. In this non-genetic PD group, the types of cancer appearing with higher prevalence in PD patients were skin cancer (4.60%), prostate cancer (2.69%), breast cancer (0.98%), urinary tract cancer (0.66%), and lymphoma (0.39%), with other types occurring with frequencies of <0.5%. Concerning the genetic subgroups, 8.04% of the *LRRK2* mutation carriers had a positive cancer history (16 patients out of 199). The types of cancer more commonly encountered in this group were, again, skin cancer (4.52%) and lymphoma (two patients, 1.01%), while other types occurred in unique cases (percentages of 0.5%). Patients harboring *GBA1* mutation had a rather low percentage of a positive history of cancer (6 patients out of a total of 107, prevalence 5.61%). Even in this small subgroup, skin cancer accounted for a percentage of 1.87%, as did lymphoma, while prostate cancer accounted for a unique patient (0.93%). Interestingly, 3 (18.75%) out of 16 *PRKN* mutation carriers with PD had a positive cancer history, which aligns with previous data of an increased risk of cancer in these patients [21]. Three different cancer types appeared in one particular *PRKN*-carrier PD patient, which again highlights the high risk of cancer in *PRKN* carriers. The rest of the genetic subgroups (*SNCA* or *PINK1* carriers) accounted for just a small number of patients with a medical history of cancer; thus, they were not able to point to any particular cancer predisposition concomitant with harboring such mutations. No cases of cancer were reported in the dual *LRRK2/GBA1* mutation carriers.

**Figure 1:**
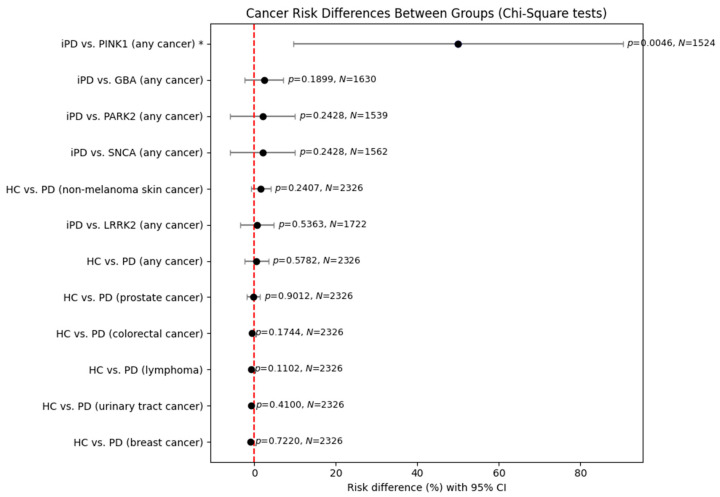
Comparison of cancer risk between healthy control individuals and PD patients using chi-square test. Statistically significant results require *p*-value < 0.05. Asterisk * marks significant results (*p* < 0.05). CI, confidence interval.

**Figure 2:**
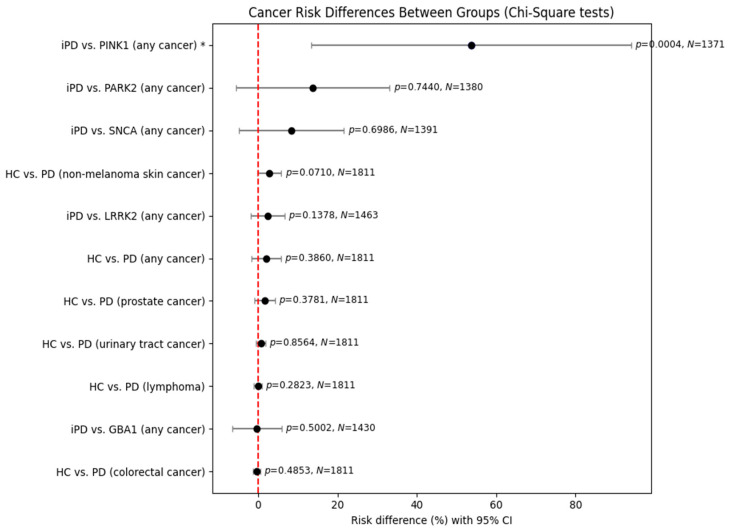
Comparison of cancer risk between healthy control individuals and PD patients of male gender using chi-square test. Statistically significant results require *p*-value < 0.05. Asterisk * marks significant results (*p* < 0.05). CI, confidence interval.

**Figure 3:**
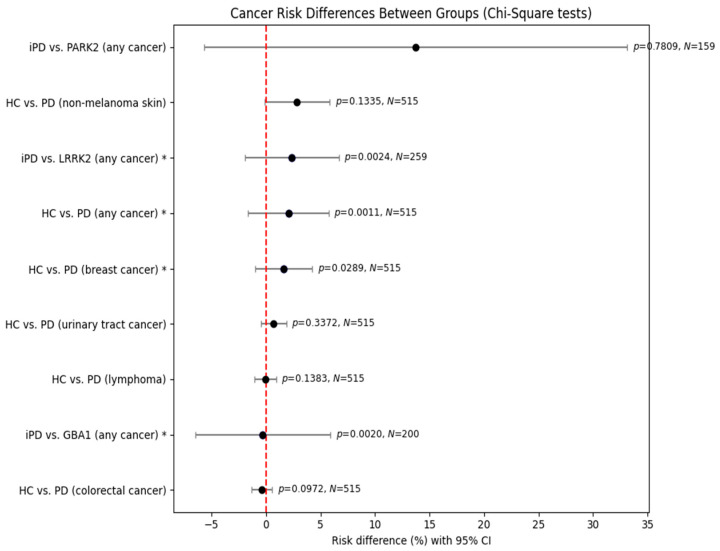
Comparison of cancer risk between healthy control individuals and PD patients of female gender using chi-square test. Statistically significant results require *p*-value < 0.05. Asterisk * marks significant results (*p* < 0.05). CI, confidence interval.

In light of the above findings, we further undertook a statistical analysis in order to assess putative differences between healthy controls and PD patients regarding not only the risk of cancer of any type but also the risk of the most frequent cancers noted in their medical histories (Figure 1). No statistically significant results were observed, except a significant risk for *PINK1* carriers versus idiopathic PD patients. However, this result has its limitations because of the small (just one unique patient) sample size of *PINK1* carriers. In order to examine gender differences between the above groups, we conducted a further statistical analysis, dividing our data on patients by sex (Figure 2 and Figure 3).

The previously observed results were reproduced in the analysis of male patients. Again, no statistically significant results were obtained, except a significant risk for *PINK1* carriers versus those with idiopathic PD, with its previous limitation of a small sample size.

In comparisons between healthy controls (HCs) and female Parkinson’s disease (PD) patients, PD was associated with significantly increased risks of any cancer (χ^2^ = 10.667, *p* = 0.0011) and breast cancer (χ^2^ = 4.771, *p* = 0.0289). No significant differences were observed for non-melanoma skin cancer, colorectal cancer, urinary tract cancer, or lymphoma.

In comparisons of iPD and genetic PD subgroups, *LRRK2* carriers demonstrated a significantly increased cancer risk. The analysis of *GBA1* carriers also yielded statistical significance (χ^2^ = 9.531, *p* = 0.0020); however, the estimated effect size was close to zero (95% CI: −6.49% to +5.91%), suggesting that the result reflects heterogeneity rather than a consistent increase or decrease in cancer risk. Thus, while *GBA1* carriers differed statistically from those with iPD in terms of cancer occurrence, no directional risk association could be established. By contrast, *PRKN/PARK2* mutation carriers did not differ from those with iPD (χ^2^ = 0.077, *p* = 0.7809).

## 4. Discussion

In the present study we assessed the association of cancer with idiopathic and genetic PD in the PPMI cohort, and we further analyzed the frequency of certain forms of neoplasms in this population. Our results support a similar prevalence of cancer history in the PPMI PD cohort and the HC group. The most common type of cancer was skin cancer, a result supported by previous literature data [14,18]. Other relatively common neoplasms in the PD cohort were prostate and breast cancer, while colorectal and urinary tract cancer accounted for approximately 5%. Lymphoma was the more prevalent hematological malignancy, whereas leukemia and multiple myeloma followed in terms of frequency. Published epidemiological data indicate an association between PD and cancer [19,22]. Overall, individuals with Parkinson’s disease (PD) tend to show a lower incidence of several cancers, and some reports even suggest that cancer patients are less likely to develop PD—a phenomenon often described as inverse comorbidity. Still, this protective trend does not apply uniformly across all malignancies. Certain cancers, including breast and brain tumors, malignant melanoma, and non-melanoma skin cancers, have been observed at higher rates in PD populations. In contrast, risks for bladder, prostate, and colorectal cancers appear reduced in comparison to the general population [18,23]. Both genetic influences and environmental exposures, such as smoking, may significantly contribute to the interplay between these two seemingly unrelated conditions [22].

One of the major strengths of this study is the use of the PPMI database, which offers a large participant pool and standardized methods of data collection. In addition, the idiopathic PD group consisted primarily of newly diagnosed, untreated patients with less than two years of disease duration at baseline. This design minimizes the chance that prior PD therapies—such as L-Dopa, rasagiline, or amantadine—might confound potential associations between PD and cancer. On the other hand, despite the relatively high number of participants in the PPMI cohorts, the overall PD sample size is modest compared to those for large epidemiological studies based on national registries. The vast majority of PPMI PD participants identify themselves as white, limiting the generalizability of the results to other racial or ethnic groups. Furthermore, because the PPMI database depends largely on medical histories reported by patients and their caregivers, certain details may be incomplete or underreported. This contrasts with national health registries, where information is systematically recorded by healthcare professionals during routine clinical evaluations. Moreover, most statistical comparisons failed to reach significance, mainly due to the size of subgroups compared to HCs.

We should also highlight a possible selection bias of not including many HCs with a previous cancer history as such participants might decide not to participate in a longitudinal study. A notable advantage of the PPMI cohort is its extensive representation of genetic PD cases, whereas large-scale epidemiological studies typically lack such detail and concentrate primarily on idiopathic PD, regardless of underlying genetic status.

Based on PPMI data, it is evident that, beyond the well-documented association between idiopathic PD and cancer, individuals harboring mutations in PD-related genes also demonstrate an elevated risk of developing malignancies. This finding may have important implications for both clinical surveillance and therapeutic strategies. Moreover, a deeper understanding of the shared molecular mechanisms that drive the onset of PD and cancer could aid in the pursuit of effective disease-modifying treatments.

Mutations in *LRRK2* are among the most frequent genetic causes of both sporadic and familial PD. These variants promote the aggregation of α-synuclein into Lewy bodies and tau inclusions while also influencing mitochondrial activity and inflammatory processes within the nervous system. In addition to its presence in neural tissue, *LRRK2* is expressed in peripheral organs and systems—including blood cells, lungs, the gastrointestinal tract, and breast tissue—providing a possible explanation for the higher incidence of hematologic, brain, colon, and breast cancers observed in individuals with *LRRK2*-related PD [24,25].

Our analysis of PPMI data indicated that among *LRRK2* mutation carriers, the cancers most frequently observed were non-melanoma skin tumors and hematologic malignancies such as lymphoma, leukemia, and multiple myeloma. However, evidence from the broader literature remains inconsistent [23,25,26]. Some reports suggest that individuals with the G2019S LRRK2 variant face an elevated risk of cancers of the blood, brain, and breast [27]. In contrast, a meta-analysis by Lee and colleagues (2022) concluded that *LRRK2*-associated PD was linked to a lower likelihood of lung and colorectal cancers but a greater risk of brain cancer and melanoma [18]. Interestingly, melanoma rates in PD patients carrying the heterozygous p.Gly2019Ser mutation were found to be comparable to—or even higher than—those in patients with idiopathic PD [28]. On the other hand, findings from Ruiz-Martinez and collaborators reported no clear association between the R1441G or G2019S *LRRK2* variant and any specific cancer type [27].

We found only a limited number of *GBA1* PD carriers with a positive cancer history (mainly skin cancer (including melanoma) and lymphomas). Regarding *GBA1* PD, most literature evidence on its association with cancer derives from studies in Gaucher disease (GD) patients carrying homozygous or compound heterozygous *GBA1* mutations [29]. An elevated frequency of cancers—including melanoma, non-melanoma skin tumors (such as basal cell and squamous cell carcinoma), hematologic malignancies (notably lymphoma and multiple myeloma), and glioblastoma, as well as breast, pancreatic, prostate, thyroid, colon, lung, renal, and soft tissue cancers—has been documented in patients with Gaucher disease (GD) [29,30]. Supporting this, Taddei and colleagues reported that in a cohort of 367 Ashkenazi Jewish GD patients (54% homozygous for the N370S *GBA1* mutation), long-term follow-up revealed heightened risks of both cancer in general and hematologic malignancies in particular [31].

Several mechanisms have been proposed to account for the cancer susceptibility associated with *GBA1* mutations. The accumulation of undegraded lipid substrates such as β-glucosylceramide (GlcCer) and β-glucosylsphingosine (GlcSph) has been implicated, particularly through their effects on immune system activity. Sphingolipid derivatives of GlcCer and GlcSph are thought to increase vulnerability to the hematological malignancies seen in GD. Moreover, sphingosine-1-phosphate has recognized oncogenic properties, including the promotion of inflammation, immune cell trafficking, and tumor growth [32].

In addition, glucocerebrosidase deficiency is linked to impaired autophagy, a critical regulator of cellular homeostasis. Defective autophagy contributes to tumorigenesis via mitochondrial dysfunction and elevated production of reactive oxygen species [33]. Finally, protein misfolding and impaired α-synuclein clearance, consequences of *GBA1* mutations in PD, may further predispose affected individuals to malignancy by activating oncogenic pathways [34].

Regarding rarer genetic forms of PD, *SNCA* carriers, albeit rare, do not show a specific trend of association with any cancer type (two cases of colon and prostate cancer in elderly males might be considered coincidental). In our study, 3/16 *PRKN* carriers had a positive cancer history [mostly skin cancer (non-melanoma) and other types like breast, prostate, or colorectal cancer]. Additionally, the only *PINK1* carrier in the PPMI study had a history of lymphoma and thyroid cancer. Earlier studies suggest that recessive PD-related genes, particularly those linked to mitochondrial activity and oxidative stress regulation, may contribute to the pathogenesis of both PD and cancer. Reduced or impaired expression of the Parkin gene (*PRKN*), which is recognized for its role in tumor suppression, can facilitate tumorigenesis and promote the growth of certain cancers. Evidence further supports *PRKN* as a critical tumor-suppressor factor in cancer development [35].

The role of *PINK1* appears to be context-dependent, with findings indicating it can function either as an oncogene or as a tumor suppressor depending on cellular conditions. Additionally, overexpression of DJ-1 has been reported in various malignancies, including lung cancer, breast cancer, and melanoma, suggesting its involvement in oncogenic pathways [15].

Finally, it appears that there might occur a sex dimorphism regarding the association of PD with cancer. In female patients, when assessed separately, PD was associated with significantly increased risks of any cancer and breast cancer, but there were no differences in other types of cancer including non-melanoma skin cancer, colorectal cancer, urinary tract cancer, and lymphoma. Notably, female PD patients harboring pathogenic *LRRK2* variants demonstrated a significantly increased overall cancer risk compared to female iPD patients as well.

The distinctive contribution of this study lies in its examination of genetic forms of PD—including carriers of *LRRK2*, *GBA1*, *SNCA*, *PRKN*, and *PINK1* mutations—in relation to cancer risk. The existing literature on this topic is sparse; most reports addressing *LRRK2* are limited in scope, while studies of *GBA1* have largely concentrated on patients with Gaucher disease rather than PD cohorts carrying heterozygous pathogenic variants [32]. Previous research on recessive PD genes was also scarce, although there are hints of an etiological connection between the two conditions.

## 5. Conclusions

In the current study we evaluated the association of idiopathic and genetic PD forms with a positive history of neoplasms in a large study cohort. The outcomes of our assessments are relevant due to the complex interaction between neurodegeneration and cancer and due to the necessity of evaluating an extensive phenotype of genetic parkinsonism far beyond the motor symptom spectrum.

## Figures and Tables

**Table 1:** Demographic and clinical data on PD patients with cancer.

Feature	
Race	97.68% White, Asian 1.75%, Other 0.58%
Sex	64.91% Male, 35.09% Female
Cancer antecedent to PD	70.76%
Age at cancer diagnosis	63.20 ± 11.34 years old (Mean ± SD)
Age at PD diagnosis	66.25 ± 8.53 years old (Mean ± SD)

**Table 2:** Demographic and clinical data on healthy control individuals with cancer.

Feature	
Race	97.22% White, Other 2.78%
Sex	61.11% Male, 38.89% female
Age at cancer diagnosis	62.61 ± 11.45 years old (Mean ± SD)

**Table 3:** Prevalence of cancer types among HCs and PD patients.

Groups	HC	Total PD	iPD	*LRRK2*	*GBA1*	*SNCA*	*PRKN*	*PINK1*	*LRRK2/* *GBA1*
Total number	438	1888	1523	199	107	39	16	1	3
All types of cancer (Non-melanoma)	36 (8.22%)	171 (9.06%)	143 (9.39%)	16 (8.0 4%)	6 (5.61%)	2 (5.13%)	3 (18.75%)	1 (100%)	0
Other skin	25 (5.71%)	83 (4.40%)	70 (4.60%)	9 (4.52%)	2 (1.87%)	0	2 (12.50%)	0	0
Brain	0	1 (0.05%)	1 (0.07%)	0	0	0	0	0	0
Lung	1 (0.23%)	4 (0.21%)	3 (0.20%)	1 (0.50%)	0	0	0	0	0
Breast	3 (0.68%)	16 (0.85%)	15 (0.98%)	0	0	0	1 (6.25%)	0	0
Gynecological	0	4 (0.21%)	4 (0.26%)	0	0	0	0	0	0
Prostate	10 (2.28%)	45 (2.38%)	41 (2.69%)	1 (0.50%)	1 (0.93%)	1 (2.56%)	1 (6.25%)	0	0
Leukemia	0	4 (0.21%)	3 (0.20%)	1 (0.50%)	0	0	0	0	0
Lymphoma	0	11 (0.58%)	6 (0.39%)	2 (1.01%)	2 (1.87%)	0	0	1 (100%)	0
Multiple myeloma	0	2 (0.11%)	1 (0.07%)	1 (0.50%)	0	0	0	0	0
Colorectal	0	8 (0.42%)	5 (0.33%)	1 (0.50%)	0	1 (2.56%)	1 (6.25%)	0	0
Pancreas	0	1 (0.05%)	1 (0.07%)	0	0	0	0	0	0
Liver	0	0	0	0	0	0	0	0	0
Stomach	0	1 (0.05%)	1 (0.07%)	0	0	0	0	0	0
Head and neck	0	4 (0.21%)	4 (0.26%)	0	0	0	0	0	0
Thyroid	1 (0.23%)	6 (0.32%)	5 (0.33%)	0	0	0	0	1 (100%)	0
Urinary tract	1 (0.23%)	10 (0.53%)	10 (0.66%)	0	0	0	0	0	0
Other	4 (0.91%)	4 (0.21%)	4 (0.26%)	0	0	0	0	0	0

## Data Availability

The dataset used in this analysis was accessed through the PPMI database and can be obtained upon reasonable request following the required registration process.

## Abbreviations

The following abbreviations are used in this manuscript:

*SNCA*	Alpha Synuclein
*LRRK2*	Leucine-Rich Repeat Kinase 2
*PRKN*	Parkin
*PINK1*	PTEN-induced kinase 1
*GBA1*	Glucocerebrosidase gene
